# Multimodal Cardiac Imaging in the Assessment of Patients Who Have Suffered a Cardioembolic Stroke: A Review

**DOI:** 10.3390/jcdd11010013

**Published:** 2023-12-31

**Authors:** Elizabeth Hui En Thong, William K. F. Kong, Kian-Keong Poh, Raymond Wong, Ping Chai, Ching-Hui Sia

**Affiliations:** 1 Department of Medicine, National University Health System, Singapore 119228, Singapore; elizabeth.thong@mohh.com.sg; 2 Department of Cardiology, National University Heart Centre Singapore, Singapore 119074, Singapore; william_kong@nuhs.edu.sg (W.K.F.K.); mdcpkk@nuhs.edu.sg (K.-K.P.); raymond_cc_wong@nuhs.edu.sg (R.W.); ping_chai@nuhs.edu.sg (P.C.)

**Keywords:** cardioembolic, stroke, imaging, echocardiogram, nuclear imaging, computed tomography, magnetic resonance imaging

## Abstract

Cardioembolic strokes account for 20–25% of all ischaemic strokes, with their incidence increasing with age. Cardiac imaging plays a crucial role in identifying cardioembolic causes of stroke, with early and accurate identification affecting treatment, preventing recurrence, and reducing stroke incidence. Echocardiography serves as the mainstay of cardiac evaluation. Transthoracic echocardiography (TTE) is the first line in the basic evaluation of structural heart disorders, valvular disease, vegetations, and intraventricular thrombus. It can be used to measure chamber size and systolic/diastolic function. Trans-oesophageal echocardiography (TOE) yields better results in identifying potential cardioembolic sources of stroke and should be strongly considered, especially if TTE does not yield adequate results. Cardiac computed tomography and cardiac magnetic resonance imaging provide better soft tissue characterisation, high-grade anatomical information, spatial and temporal visualisation, and image reconstruction in multiple planes, especially with contrast. These techniques are useful in cases of inconclusive echocardiograms and can be used to detect and characterise valvular lesions, thrombi, fibrosis, cardiomyopathies, and aortic plaques. Nuclear imaging is not routinely used, but it can be used to assess left-ventricular perfusion, function, and dimensions and may be useful in cases of infective endocarditis. Its use should be considered on a case-by-case basis. The accuracy of each imaging modality depends on the likely source of cardioembolism, and the choice of imaging approach should be tailored to individual patients.

## 1. Introduction

Up to 26 million people are diagnosed as having suffered a stroke every year, constituting the second most common cause of mortality [1]. Strokes also carry significant risks of morbidity and functional disability [1]. Cardioembolic strokes account for up for 20–25% of all ischaemic strokes, with their incidence increasing with age (14.6% of patients <65 years old, and 36% of patients >85 years old) [2,3,4]. Affected patients usually present with a sudden-onset neurological deficit that is maximal at onset and a decreased consciousness level at onset, often affecting the cerebral cortex, and they may present with cortical signs (aphasia and visual field deficits) and concurrent cerebral and systemic emboli [3,5].

The diagnosis of cardioembolic stroke is based on a few classification systems. The TOAST criteria (Trial of Org 10172 in Acute Stroke Treatment) focuses on classifying the cause of stroke into a single aetiology, with non-overlapping definitions [5]. However, patients often have multiple overlapping risk factors for different subtypes of ischaemic stroke rather than a single causative factor. Other classification systems were hence developed to complement the TOAST system, with the CCS (Causative Classification of Stroke) [6] system serving as an attempt to better classify patients with undetermined aetiologies and the ASCOD (atherosclerosis, small-vessel disease, cardiac pathology, other causes, or dissection) [7] system serving as an attempt to reduce the number of strokes of undetermined cause via the inclusion of all possible aetiologies of stroke [8]. These systems acknowledge that patients may not have a single, clear-cut cause of their stroke, instead giving the probability of each individual mechanism in contributing to a stroke. In all three classification systems, imaging plays a crucial role.

Cardioembolic stroke is defined as a stroke secondary to an embolus from an identified cardiac source occurring without significant arterial stenosis [5]. It occurs when a cardiac source potentiates a component of Virchow’s triad: endothelial injury, stasis, and hypercoagulability [9]. Risk factors for cardioembolic stroke include factors potentiating thrombus formation (atrial fibrillation (AF), left-atrial enlargement, acute myocardial infarction, aortic arch atheroma, cardiomyopathies, and cardiac tumours), defects of the atrial septum (patent foramen ovale and atrial septal aneurysm), and valvulopathies (infective endocarditis, prosthetic valves, and mitral and aortic valvulopathies). Whilst AF is the most common cause of cardioembolic stroke, other cardioembolic sources necessitate dedicated cardiac imaging. The detection of potential cardioembolic sources of stroke is crucial and can significantly affect management, including the initiation of anticoagulation, antibiotics and surgical intervention. As such, the early and accurate identification of the source of a cardioembolic stroke is paramount for the timely initiation of treatment, preventing recurrence, and reducing stroke incidence [10].

In this paper, we will discuss the multimodal cardiac imaging techniques used in the assessment of patients who have suffered a cardioembolic stroke, with cardiac imaging also potentially improving diagnostic accuracy. The accuracy, sensitivity, and specificity of each imaging modality depend on the specific cardioembolic sources of stroke. As such, we will provide both an overview of imaging techniques as well as a detailed examination of each imaging modality in relation to its use in identifying an individual cardioembolic source.

## 2. Materials and Methods

On 2 October 2023, we conducted a comprehensive search on Pubmed, Scopus, Embase, and MEDLINE for studies pertaining to the following terms: (cardiovascular imaging OR cardiac imaging OR echocardiogram OR echocardiography OR computed tomography OR magnetic resonance imaging OR positron emission tomography OR molecular OR single-photon emission computed tomography) AND (cardioembolic OR stroke OR infarct OR acute ischaemic infarct OR transient ischaemic attack). We conducted further searches for use of imaging (as per the above terms) in the assessment of individual cardiac risk factors for stroke, employing the following terms: (atrial fibrillation OR arrhythmia OR sick sinus syndrome; left atrial thrombus OR left atrial appendage OR left atrial enlargement; cardiac tumours OR cardiac masses OR cardiac myxoma OR papillary fibroelastoma; HF OR congestive HF; acute myocardial infarction OR myocardial infarction OR heart attack; intracardiac thrombus OR cardiac thrombus OR left ventricular thrombus; patent foramen ovale; infective endocarditis OR valvular abscess OR valvular infection OR vegetation OR perforation; mitral stenosis OR mitral regurgitation OR mitral valve prolapse OR mitral annulus calcification; aortic stenosis OR aortic regurgitation OR aortic arch atheroma OR aortic plaque; prosthetic valve OR valve thrombosis; cardiomyopathy). Inclusion criteria included the following: study type—observational, prospective, cohort, cross-sectional, comparative, randomised control trial, systematic review, review, or guideline; studies that used quantitative methodology; and articles published in English. Exclusion criteria included studies that used qualitative methodology and/or animal models and that were published in a language other than English. Our focus was on studies that reviewed cardiovascular imaging modalities that can be used in the assessment of patients with cardioembolic stroke, especially based on the specific cardiac risk factor involved. Screening of studies was initially based on titles and abstracts, and we also manually identified additional relevant articles through an extensive search of references in literature reviews. Subsequently, all pertinent articles underwent a thorough full-text review.

## 3. Discussion

### 3.1. Overview of Cardiac Imaging in Cardioembolic Stroke

#### 3.1.1. Transthoracic Echocardiography (TTE)

Echocardiography is the mainstay of cardiac evaluation for cardioembolic stroke, with all associated guidelines recommending echocardiography in the workup of cardioembolic stroke [11,12,13]. However, whether transthoracic echocardiography (TTE) or transoesophageal echocardiography (TOE) should be used as the first-line treatment is not clear in the American Heart Association, American Stroke Association, and European Society of Cardiology guidelines. The guidelines issued by the European Association of Echocardiography and the European Stroke Organisation instead recommend that both TTE and TOE can be used in the evaluation of a potential cardioembolic stroke [14,15].

TTE allows for the identification and imaging of structural heart disorders, valvular disease, vegetations, and intraventricular thrombus and can be used to measure chamber size and systolic and diastolic function (Table 1) [16,17]. It is readily available, non-invasive, and cheaper than TOE [18]. TTE is also a first-line treatment used to identify infective endocarditis (sensitivity 62–79%), with TOE (sensitivity 85–90%) being used for indeterminate TTE findings and/or abscesses [19]. TTE is also highly sensitive and specific (with corresponding values of 96% and 90%, respectively, without contrast) for LV thrombus detection [20]. TTE with contrast can also improve image visualisation, with Kurt et al.’s prospective cohort study finding that contrast use decreased the percentage of technically difficult studies from 86.7% to 9.8% (*p* < 0.0001) and uninterpretable studies from 11.7% to 0.3% (*p* < 0.0001) [21]. This resulted in the avoidance of additional diagnostic procedures for 32.8% of patients and affected medication choices for 10.4% of patients [21]. Three-dimensional echocardiography can also provide multiplanar details on chamber size, cardiac mechanics, and complex geometrical shape volumes [22,23,24].

Limitations: Whilst TTE is readily available, non-invasive, and cheaper than other imaging modalities, its use entails inter-operator variability and potentially limited acoustic windows depending on body habitus [25,26,27]. It may also provide limited views of apical lesions due to the proximity of the left-ventricular apex to the chest wall, offer limited visualisation of the mitral valve, have difficulty differentiating mass mimics from true masses, and provide limited evaluations of pericardial disease [26]. In view of these limitations, additional computed tomography (CT) or cardiac magnetic resonance imaging (CMR) can be considered to provide a comprehensive cardiac assessment.

#### 3.1.2. Transoesophageal Echocardiography (TOE)

Transoesophageal echocardiography (TOE) is the gold standard for detecting high-risk and potential cardioembolic sources of stroke, with a diagnostic yield of 40–60% [28,29,30]. It gives accurate information on valve vegetations, pulmonary veins and aortic arch and ascending aorta, the left atrium and left-atrial appendages, and the intra-arterial septum and can identify high-risk causes of stroke such as left-atrial flow velocity < 40 cm/s, thrombi in the left-atrial cavity/left-atrial appendage, aortic thrombi or plaques ≥ 4 mm, and spontaneous echo contrast (Table 1) [16,28,31]. However, its role in the acute evaluation of ischaemic stroke is not as well established as that of TTE, as it is easier and faster to obtain a transthoracic echocardiogram [32,33].

De Castro et al.’s prospective cohort study found that 40% of previously classified undetermined strokes were re-classified as cardioembolic strokes using TOE, resulting in 12% of patients being shifted from antiplatelet to anticoagulation therapy [34]. With 26% of secondary prevention management being modified by TOE results, the early use of TOE may have a significant role in identifying risk factors for recurrent stroke and affecting management [34]. The CONTEST (Comparative Effectiveness Study of Transthoracic and Transesophageal Echocardiography in Stroke) study found that TOE findings resulted in a change of stroke mechanism in 11.5% of patients, with an increase in the number of strokes classified as cardioembolic and a reduced number classified as cryptogenic [35]. Notably, Ulrich et al. found that patients with multivessel strokes exhibited a lower number of possible cardioembolic sources according to TOE compared to those with single-vessel or lacunar strokes [36]. This suggests that TOE may be able to aid in classification for patients for whom routine investigations yield unsatisfactory results but that it may also have limited use for patients who have suffered a multivessel stroke.

TOE also has a role for patients without atrial fibrillation as well. De Castro et al. found that 40% of patients with cardioembolic stroke were in sinus rhythm, highlighting the importance of echocardiographic evaluation for other high-risk cardioembolic sources for patients suspected to have suffered a cardioembolic stroke [34].

However, the usefulness of TOE across different age groups remains controversial. Whilst the proportion of each aetiology varies based on age, there is no specific age-specific recommendation regarding the need for TOE, and the decision to carry out echocardiography is instead based on potential aetiology and risk factors [37]. Some studies, including the Find-AF_RANDOMISED_ study, have found that TOE is useful for younger stroke patients due to an increased prevalence of atrial septal abnormalities [18,38,39,40] and for patients with undetermined stroke [28,39]. Other studies have found that TOE offers significant benefits regardless of age [41,42,43,44]. TOE may also provide an advantage for assessing older patients: complex aortic plaques and regional wall motion abnormalities are more commonly found in older patients, constituting a major risk factor for recurrent stroke [18,40,44]. Overall, guidelines recommend echocardiography for patients with suspected embolic stroke and without contraindications for oral anticoagulation, as this type of stroke’s diagnosis affects treatment, which tends to be administered to younger patients [37]. In comparison, guidelines recommend TTE for patients with at least one established cardiovascular risk factor, which is more common in older age groups [37]. The choice of TOE vs. TTE should be made on an individual level and based on clinical suspicion.

Limitations: The main limitation of TOE is its semi-invasive nature, meaning that it cannot be used for patients with poor systemic condition, who are not fit to undergo light-moderate sedation, and with decreased consciousness [44]. In addition, it is more resource-heavy and expensive compared to TTE, with minor procedural risks [45].

#### 3.1.3. TTE vs. TOE

Compared to TTE, TOE has greater sensitivity and specificity with respect to identifying cardioembolic sources of stroke (thrombosis, contrast, aortic lesions, PFO, atrial septal aneurysm, mitral vegetation, and left-atrial appendage thrombi) (Table 1) [27,46]. In clinical practice, TTE is more frequently performed compared to TOE, and TOE is not usually performed in the presence of a normal transthoracic echocardiogram unless the suspicion for a false-negative transthoracic echocardiogram is high. 

The Find-AF_RANDOMISED_ prospective multicentre randomised controlled trial (*n* = 402) found that TOE resulted in a change in therapy for 9.0% of patients, whereas TTE only resulted in a change in therapy for 0.3% of patients [18]. Similarly, De Bruijn et al.’s prospective cohort study (*n* = 231) evaluated the role of TTE vs. TOE in the management of stroke and TIA and found that 39% of potential cardioembolic sources of stroke were observed solely via TOE and not via TTE across all ages [41]. The CONTEST prospective multicentre study found that TOE was better than TTE at identifying treatment-relevant findings (18.9% vs. 14.1%, *p* < 0.001), and this was especially pronounced for younger patients < 60 years old [35]. Notably, the CONTEST study found that the diagnostic yield of TOE in identifying cardioembolic sources when PFO was excluded was <1%, likely due to modern TTE technologies [35]. With PFO being a more common cause of cryptogenic stroke in younger patients, clinicians can consider the role of upfront TOE in assessing younger patients [35].

Both TTE and TOE are used in the identification of infective endocarditis. TTE has a sensitivity of 62–79%, and TOE has a sensitivity of 85–90% [19]. TOE is better for evaluating leaflet tears and abscesses [19]. In the detection of mitral and aortic valve prostheses, TOE is superior to TTE (TOE sensitivity: 80–90%; TTE sensitivity: 20–40%) [19]. In the detection of abscesses, TOE has higher sensitivity than TTE (87% vs. 28% respectively) but lower specificity (95% vs. 98%, respectively) [19].

Overall, TOE offers greater yields in identifying potential cardioembolic sources of stroke compared to TTE, with the classification of stroke aetiology changing for over 10% of patients [35]. Using TOE instead of TTE should be strongly considered in evaluations of cardioembolic sources of stroke, especially for patients with undetermined strokes, with this decision also influencing the evaluation of atrial septal defects in younger patients and complex aortic atheroma in older patients.

#### 3.1.4. Cardiac Computed Tomography (CT) and Cardiac Magnetic Resonance Imaging (CMR)

Compared to echocardiography, cardiac CT and CMR offer better soft tissue characterisation, high-grade anatomical information, spatial and temporal visualisation, and image reconstruction in multiple planes (Table 1) [47]. They also provide information on associated complications of the discussed disease, including pericardial effusion, valvular dysfunction, and complications of IE [48,49]. Cardiac CT can be used in complex valvular prosthesis cases and is a good alternative for those for whom CMR is contraindicated (e.g., in cases involving some implantable cardiac devices) [50].

CMR excels in visualizing valvular flow patterns and function, chamber volumes, and myocardial function [51,52]. It avoids the limitations of operator dependence and reliance on body habitus. Especially with contrast, it is more accurate than TTE and TOE for the diagnosis of LV thrombus and can also identify structural features that increase risk for LV thrombus, such as myocardial scar burden/infarct size and distribution (Table 1) [25,53,54,55,56]. CMR with phase contrast velocity mapping can be used to quantify and precisely locate regurgitant jets more efficiently than echocardiography and can be especially useful in the suboptimal quantification of regurgitant jets via TOE [57]. Late-gadolinium enhancement CMR (LGE-CMR) enhances the ability to detect and characterise LV thrombi, fibrosis, and specific causes of heart failure (HF) such as infiltrative and inflammatory cardiomyopathies [9,58]. CMR can assess aortic plaque structures and instability, with three-dimensional-multi-contrast MRI providing further details of plaque characteristics and morphology, including size, the presence of intraplaque haemorrhages, and superimposed thrombi [22,59]. Currently, there are insufficient data on CMR’s diagnostic role in IE [60,61].

When comparing the two, cardiac CT and CMR have comparable diagnostic performance in visualising LA appendage thrombus and high-grade valve disease/calcifications, with both being inferior to TOE in imaging valvular AF, mitral valve prolapse, and high-grade valve disease/calcifications [62]. Cardiac CT is superior to CMR in terms of imaging aortic dissection, aortic aneurysm, and complex aortic plaques [62]. In comparison, CMR is superior to CT in terms of imaging LA characteristics (volume, morphology, and function), LV thrombus, non-thrombotic masses (e.g., myxoma and cardiac tumours), LV aneurysms, cardiomyopathies, aortitis, wall hypo-/akinesia, and reduced ejection fractions (Table 1) [62].

Limitations: Notably, the choice of imaging is largely influenced by resource allocation and availability. Generally, CT is more widely available, provides results faster, and yields a faster scan, allowing for large infarctions or mass effects to be seen and acted upon [63]. CMR is more sensitive to smaller infarctions but may not be as widely available; is more costly; and takes a longer time to perform. CT costs 3 times more than TTE, with CMR costing 5.5 times more than TTE [64]. Radiation exposure remains a relevant issue in stroke CT, especially if contrast angiography and perfusion-CT datasets are acquired [63]. Whilst there is a low rate (0.1%) of adverse events for gadolinium-enhanced contrast used in CMR, it is relatively contraindicated for patients with poor renal function due to an increased risk of nephrogenic systemic fibrosis and in those that are pregnant [65]. Iodine contrast is relatively contraindicated for severe renal impairment due to an increased risk of contrast-induced nephropathy, active thyrotoxicosis, and multiple allergies [65]. Cardiac CT also cannot be used to measure flow velocity, perform hemodynamic assessment, or conduct regurgitant quantification [47]. The accuracy of both CMR and cardiac CT is reduced in patients with high heart rates, with its image quality relying on the patient having a heart rate of <60 beats per minute [58]. The use of CMR and CT in the evaluation of patients should be considered on a case-by-case basis.

#### 3.1.5. Nuclear Imaging

The primary role of nuclear imaging lies in assessing LV perfusion (ischemia and infarct), function, and dimensions [66]. It can provide key information on fundamental pathophysiological mechanisms and molecular processes of cardiovascular disorders that increase the risk of cardioembolic stroke, such as cardiomyopathies, infiltrative and inflammatory heart diseases, and complications of arrhythmias and HF (Table 1) [66]. Position emission tomography (PET) and single-photon emission computed tomography (SPECT) provide metabolic and functional information that can be used to increase diagnostic accuracy and the localisation of lesions [67]. It is also used in the diagnosis and staging of cardiac tumours and for diagnosing IE via the higher metabolic activity in inflammatory tissue, especially in difficult cases [53,68] can also identify plaque inflammation and hypermetabolism, which place the patient at higher risk of suffering a plaque rupture [40,65,66]. PET tracers can also be used to track inflammation, hypoxia, neoangiogenesis, and calcification, which are potential markers of plaque rupture [67]. As such, Rominger et al. found that radioisotope uptake in major arteries was a strong predictor of vascular events [69]. Whole-body PET/CT has >90% sensitivity and specificity for cancer diagnosis [70]. With both cancer as well as some cancer treatments being associated with increased thrombotic risk due to cancer-related hypercoagulability, intracardiac tumours, or intracranial arterial compression caused by brain tumours, PET/CT can be considered if an underlying malignancy is suspected [71].

Limitations: Nuclear imaging is not always readily available, and it is complex and costly [72]. Limited availability may delay the scheduling of the scan and intervention thereafter. The use of radioisotopes also carries a small risk of radiation equivalent to over 500 chest X-rays and is controversial during pregnancy [64]. SPECT costs more than 3 times TTE, with PET costing 14 times more than TTE [64]. Overall, this limits the use of nuclear imaging in cardiac imaging in the initial assessment of patients with cardioembolic stroke, especially when faster and cheaper alternatives are available and offer comparable results. Nonetheless, it may still play a role in specific situations such as systemic cancers with increased thrombotic risk, cardiac tumours, and evaluating the risk of plaque rupture.

#### 3.1.6. Computed Tomography Angiography–Aorta (CTA)

Aortic arch atheroma is a risk factor for ischaemic stroke [36,40,73]. CTA is used to evaluate the aorta and its major branches, with high-resolution helical CTA being able to identify protruding aortic plaques, including their location, size, and density [45]. CTA can also visualise the distal ascending aorta, a location not visualisable via TTE, and can also detect vascular calcification [45]. Unlike TOE, CTA cannot be used to assess plaque mobility [51,52]. CTCA-WVS (wide-volume scanning with 320-row multidetector computed tomography coronary angiography) is another imaging modality that can be considered, as it is able to identify large and complex aortic arch atheroma morphology and its association with ischaemic stroke without requiring additional contrast volume [74].

#### 3.1.7. Overall Considerations

Overall, the choice of imaging modality, including nuclear imaging, should be tailored to the individual patient and based on their specific circumstances and potential risk factors. The choice of imaging modality should be based on a comprehensive evaluation of risks and benefits, taking into consideration resource allocation, availability, and logistical, financial, and clinical factors (Table 1).

**Table 1:** Summary of main imaging modalities used in cardiac assessment of cardioembolic stroke.

	Advantages	Disadvantages
TTE	Readily available [25,26,27] Non-invasive [25,26,27] Inexpensive [25,26,27] Sensitive and specific for LV thrombus [20]	Limited views of atria and appendages [26] Inter-operator variability [25,26,27] Potentially limited acoustic windows [25,26,27]
TOE	Gold standard for detecting high-risk and potential cardioembolic sources of stroke [28,29,30] Good views of atria and appendages [16,28,31] Better for evaluating leaflet tears and abscesses in IE [19] Can be considered for patients afflicted by cryptogenic stroke who may be reclassified as cardioembolic [34,35] Can be considered for younger patients in order to search for PFO [38,39,40] 26% of secondary prevention management modified by TOE results [34]	Semi-invasive [44] Resource-heavy [45] Expensive [45] Minor procedural risks [45] Higher risk of complications in certain patient groups (high body weight, history of gastrointestinal bleed or surgery, advanced age, and oesophageal mass/stricture/varices) and patients with higher sedation risk (chronic kidney disease, cardiac disease, pulmonary disease, liver cirrhosis) [44] Role in the acute evaluation of ischaemic stroke is not well established [32,33] Inter-operator variability [45]
Cardiac CT	Better soft tissue characterisation [47] High-grade anatomical information [47] Allows for spatial and temporal visualisation and image reconstruction in multiple planes [47] Can be used to image extra-cardiac structures [47] Greater reproducibility [63] Less inter-operator variability No dependence on acoustic window Alternative to CMR in patients contraindicated for MRI [50] Yields results faster and is easier to execute than MRI [63]	Limited by resource allocation and availability [63] Radiation exposure [63] Cannot be used to measure flow velocity or perform hemodynamic assessment or regurgitant quantification [47] Accuracy reduced in cases of high heart rates [58]
CMR	Better soft tissue characterisation [47] High-grade anatomical information [47] Allows for spatial and temporal visualisation and image reconstruction in multiple planes [47] Can be used to image extra-cardiac structures [47] Greater reproducibility [63] Less inter-operator variability No dependence on acoustic window More accurate than TTE and TOE for diagnosis of LV thrombus [25,53,54,55,56] Can be used to diagnose cardiomyopathies via LGE-CMR [9,58] No radiation exposure	Limited by resource allocation and availability [63] Accuracy reduced in cases of high heart rates [58] Expensive [63] Longer duration of scan [63] Relatively contraindicated for patients with poor renal function due to an increased risk of nephrogenic systemic fibrosis and in those that are pregnant [65]
Nuclear imaging (PET, SPECT)	Allows assessment of LV perfusion (ischemia and infarct) [66] Allows assessment of chamber function and dimension [66] Reveals fundamental pathophysiological mechanisms and provides metabolic information on molecular processes [53,68] Can identify potential markers of plaque rupture [40] Can be considered in cases of suspected underlying malignancy [71]	Limited by resource allocation and availability [72] Expensive [72] Radiation exposure [64] Use is controversial during pregnancy [64]

### 3.2. Cardiac Imaging Based on Individual Sources of Cardioembolic Stroke

#### 3.2.1. Thrombus Formation

##### Atrial Fibrillation (AF), Left-atrial (LA) Dilatation, and LA Thrombus

**Figure 1:**
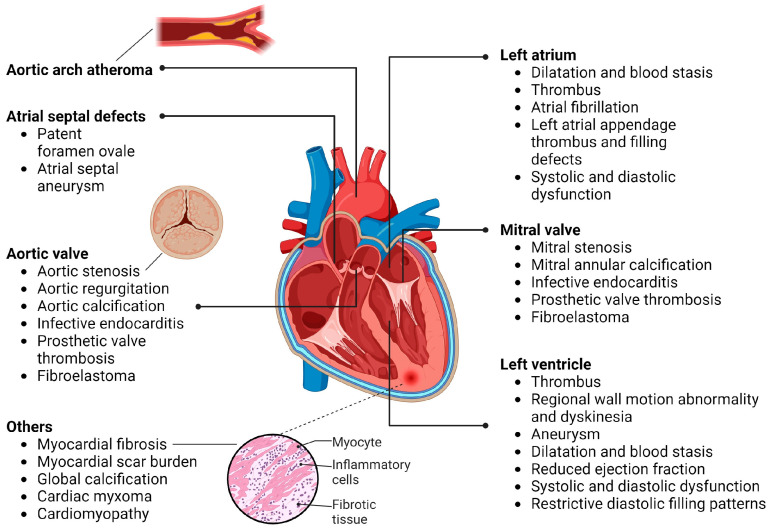
Potential causes of cardioembolic stroke.

Atrial fibrillation (AF) affects more than 33 million individuals worldwide and can increase the risk of stroke by up to 3–5 times (Figure 1) [75,76]. The prevalence of AF increases with age, affecting 9% of individuals > 80 years old [77]. AF results in reduced atrial emptying, increasing the risk of thrombogenesis and thromboembolism [78]. In addition, AF is associated with endothelial dysfunction and a hyperinflammatory response, with inflammatory molecules increasing plaque instability [79]. The early recognition and initiation of treatment for AF with anticoagulation and/or percutaneous interventions are hence crucial in stroke prevention and reducing stroke recurrence.

The focus of imaging for AF is on identifying the underlying cardiac cause of AF. TTE is recommended for the initial assessment of AF in order to identify aetiology, assess LA and LV size and function, and scan for any underlying valvular disease or rheumatic heart disease [80]. Moderate–severe LV dysfunction is also associated with an increased risk of stroke, and LA dilation is also a significant prognosticator for mortality and risk of stroke due to the loss of normal atrial geometry (Figure 1) [17,81,82,83]. Special attention should be paid to searching for LA thrombus; most commonly occurring in the LAA, it is a common cause of cardioembolic stroke and is highly associated with AF (Figure 1) [84]. LA emptying velocities < 40 cm/s are associated with higher stroke risk, and velocities < 20 cm/s are associated with LA thrombosis [85,86].

Whilst TTE can be used to assess chamber size and function, it provides limited views of the LAA and hence has low sensitivity for LA thrombus [80]. Instead, TOE is the technique of choice for detecting posterior cardiac structures, including atria and appendages, and is considered the gold-standard technique for identifying LAA thrombus (with a specificity of 100%, a sensitivity of 93%, and an accuracy of 99%, Table 2) [83]. Other TOE markers of the thrombogenic milieu include the presence of spontaneous echo contrast and LAA mechanical dysfunction (elevated pulsed-wave Doppler measurements of LAA emptying and filling velocities and early diastolic Doppler/late diastolic Doppler flow) [80]. For individuals exhibiting LAA artifacts or notable spontaneous echo contrast, the use of contrast during TEE can determine the presence of LA thrombus [87]. Real-time three-dimensional echocardiography has further enhanced this ability; it is able to distinguish between real and artefactual masses within the LA cavity and is more accurate in calculating LA volume compared to TTE [84,88].

Cardiac CT can be used to assess LAA size and morphology; however, patients with LAA stasis may have filling defects that result in a higher rate of false positives in detecting LA thrombus [87]. Whilst CMR is similarly able to provide great anatomical detail, it similarly has a higher rate of false positives due to its lower spatial resolution and its susceptibility to slow flow (Table 2) [89].

Notably, LA size in patients with AF must be interpreted with caution. Tsang et al. conducted a prospective observational study and found that LA size did not predict the risk of developing a cardiovascular event, including stroke [90]. This is largely due to AF resulting in progressive LA dilatation and advanced atrial remodelling due to tachycardia-induced atrial myopathy, regardless of left-ventricular (LV) filling pressures [91]. In addition, differences in LAA morphology also affect the risk of stroke, and for patients with a low-intermediate risk of stroke/TIA, the type of LAA morphology should be taken into account when considering anticoagulation [92].

One of the newer technologies is strain imaging (Table 2). Patients affected by stroke and AF have lower rates of peak systolic LA strain compared to patients who have not suffered a stroke [93]. A recent prospective observational study found that the global longitudinal strain (GLS) was lower in patients with acute embolism (*p* < 0.001) and allowed for the identification of patients with acute embolism (*p* < 0.0001) when compared to controls [94]. Other studies have similarly found that LAA strain has a similar predictive power for ischaemic stroke compared to the CHADS-VASC2 score [95,96], and others’ findings show that LAA strain can predict subclinical AF for patients with cryptogenic stroke [97]. GLS may also be able to predict post-stroke mortality [94]. The recently published Cardiovascular Abnormalities and Brain Lesions study evaluated LA strain and strain rate via speckle-tracking echocardiography and found that reduced positive longitudinal LA strain and negative longitudinal LA strain rate are independently associated with ischemic stroke in older adults [98]. As such, strain imaging can have a significant impact on the prediction of stroke risk and mortality and can be used to also predict subclinical AF, potentially improving risk stratification for patients afflicted by a cryptogenic stroke.

##### Acute Myocardial Infarction (AMI) and LV Thrombus

AMI is associated with increased rates of ischaemic stroke [99,100]. It also confers an increased in-hospital mortality rate of 10–20%, a 30-day mortality of 45%, and a long-term mortality of 28% [101,102,103,104]. The risk of ischaemic stroke is highest in the acute period post-AMI but remains elevated for years [105]. The causes are multifactorial. Ventricular regional wall motion abnormality and dyskinesia result in focal haemostasis, increasing the risk of mural thrombus formation (Figure 1) [106]. This risk is increased further for patients with aneurysmal dilatation of the apical or anterior ventricular wall, a lower ejection fraction, and a lower Thrombolysis In Myocardial Infarction (TIMI) score [107,108]. AMI also increases the risk of developing AF, with up to 22% of patients developing AF post-AMI [109]. Moreover, ischaemia itself results in a hypercoagulable state, with increased levels of prothrombin and fibrinopeptides, resulting in an increased risk of thrombus formation and resultant embolization [110]. The release of inflammatory cytokines, neutrophil activation, and acute phase reactants also destablilise existing plaques in the neurovasculature [111,112,113].

Echocardiography can be used to assess LV function, identify intracardiac thrombi, and search for post-AMI HF. Severe right-ventricular dysfunction, as measured via decreased right-ventricular fractional area change observed via TTE, was associated with an increased risk of cardiovascular events, including stroke (HR 2.95, 95% CI 1.76 to 4.95) [114]. TTE can be used to detect intracardiac thrombi and assess LV function, with a specificity of 85–90% and a sensitivity of 95% in detecting LV thrombus (Table 2) [9,115]. However, 10–46% of TTEs may be inconclusive due to difficulty in visualising the LV apex and may also struggle to differentiate true thrombus from thrombus mimics [116,117]. Whilst LV thrombus visualisation can be improved with contrast during TTE, contrast drugs may not be suitable for use during AMI, recent PCI, or severe HF [9,118]. In comparison, TOE is the technique of choice for detecting LV echogenic structures [80,119,120]. CT provides approximately the same specificity and sensitivity compared to TTE in the evaluation of post-AMI cardiac function and the search for intracardiac thrombi, though it is not routinely used due to posing a risk of radiation exposure [121].

CMR with contrast is the gold standard for LV thrombus detection (TTE: sensitivity—–33–40%; CMR: sensitivity—88–91%) and can also be used post-AMI to evaluate ventricular function and volumes (Table 2) [25,54,55,56]. In addition, CMR can be used to identify structural features that increase risk of LV thrombus, such as myocardial scar burden/infarct size and distribution [53]. LGE-CMR enhances the ability to detect and characterise LV thrombi, including their sizes and locations [9]. Compared to LGE-CMR, non-contrast echocardiography has a sensitivity of 33% and a specificity of 94% (with an accuracy of 82%), and contrast echocardiography has a sensitivity of 61% and a specificity of 99% (with an accuracy of 92%) in detecting LV thrombus [53]. LGE-CMR can also accurately detect LAA thrombus and right-sided thrombi, though currently there is limited evidence directly comparing CMR to TOE in the detection of LAA thrombus [53]. As such, the gold standard for LV thrombus detection is currently CMR [9].

##### Heart Failure (HF) and Cardiomyopathy

HF affects an estimated 26 million people worldwide, resulting in more than 1 million hospitalisations in the United States and Europe [122]. It carries with it a high rate of mortality and rehospitalisation [122]. Stroke rates in cases of HF range from 1–5% per year [123], with HF increasing the risk of stroke by two- to threefold, and there is a 34% prevalence of silent cerebral infarcts in patients with an ejection fraction < 20% [124,125,126]. The increased risk of stroke in HF is due to low cardiac output, dilated heart chambers, and poor contractility resulting in abnormal flow, also causing disordered regional haemostasis, platelet dysfunction, and endothelial dysfunction [127,128]. Its resultant effect on Virchow’s triad is a hypercoagulable and prothrombotic state, increasing risk of thrombosis. As such, ischaemic cardiomyopathy and dilated left-ventricular size are associated with left-ventricular thrombus formation and an increased mortality rate (Figure 1) [129]. However, a recent systematic review published in 2021 evaluating the effects of long-term oral anticoagulation in HF patients in sinus rhythm found that whilst oral anticoagulation was associated with a reduced risk of stroke, it also conveyed an increased risk of bleeding and did not reduce mortality [130]. Furthermore, there is a risk of undiagnosed AF in patients with HF [131]. The ongoing Confirm-AF (Confirm Rx Insertable Cardiac Monitor for Primary Atrial Fibrillation Detection in High-Risk HF Patients) trial is a prospective randomised, multicentre trial that aims to evaluate the utility of implantable cardiac monitors in detecting AF in HF patients with ejection an fraction > 35%, resulting in appropriate AF-related interventions [131].

With regard to cardiomyopathies, the European Cardiomyopathy Registry reports a stroke risk of 2.1–4.5% for patients with cardiomyopathy, with an incidence of AF ranging from 14–48% [57]. In some cardiomyopathies, systolic dysfunction and the resultant abnormal blood flow are considered to be the main factor potentiating increased LV thromboembolic risk (Figure 1) [132,133,134]. Structural and functional abnormalities such as atrial dilatation, atrial standstill and AF in hypertrophic cardiomyopathy [135,136,137,138,139], a dilated and aneurysmic right ventricle in arrhythmogenic right-ventricular cardiomyopathy [140,141,142], and ventricular dilatation and dysfunction in dilated cardiomyopathy [134] also result in a hypercoagulable state. Other factors that contribute include systemic factors (e.g., systemic inflammation, catecholamine surge and endothelial injury in Takotsubo syndrome [132,133], eosinophilic infiltration in hypereosinophilic syndrome [143], and increased pro-coagulant activity in peripartum cardiomyopathy [144,145]) that drive the combination of platelet and tissue factor, thereby creating a hypercoagulable state [146].

TTE is the initial modality used to identify intracardiac thrombi, reduced ejection fraction, LV regional wall motion abnormalities, and dilated LA or LV, all of which are associated with an increased risk of stroke (Table 2) [147,148]. The accuracy of TTE is increased with contrast administration, which can concomitantly identify LV thrombus [147]. A low cardiac output increases the risk of cerebral hypoperfusion, especially in vulnerable areas (e.g., watershed regions, regions supplied by deeply penetrating arteries, and regions without collateral flow) [148,149,150]. In addition to the above, TTE is also able to identify restrictive diastolic filling patterns that are associated with an increased risk of stroke [151]. Dilated LA or LV in HF can result in blood flow stasis and left-atrial and aortic spontaneous echo contrast, with LA thrombus being best imaged via TOE and LV thrombus being best imaged via TTE [148,152]. As such, for patients with cardiomyopathy who have AF or who have suffered a cryptogenic stroke, it is strongly recommended that TOE is used to look for intracardiac thrombi and spontaneous echo contrast [146]. Three-dimensional echocardiography is more accurate than TTE, as the former can image the entire LV cavity geometry and requires fewer geometrical assumptions, though it remains dependent on good acoustic windows and operator skill [147]. Stress echocardiography can assess systolic and diastolic reserve and screen for pre-clinical dilated cardiomyopathy [153].

An emerging TTE technique is speckle-tracking echocardiography, finding primary use in identifying myocardial strain, LV deformation, and chamber mechanics, though it is unable to directly estimate ejection fraction estimation (Table 2) [153]. Global longitudinal strain (GLS) can be used to evaluate LV dyssynchrony and detect subclinical LV systolic dysfunction prior to any noticeable change in LV ejection fraction [154]. It is a good marker of arrhythmias in non-ischaemic cardiomyopathy, though it has limited prognostic ability for patients with AF and limited ability for assessing patients with suboptimal acoustic windows [153,155,156]. Being a relatively new technology, there are few data on the impact of two-dimensional strain imaging on management and long-term cardiovascular outcomes [157]. However, it has been shown to have significant diagnostic and prognostic advantages [157]. As such, GLS has been incorporated into the 2017 European Association of Cardiovascular Imaging guidelines for the evaluation of left- and right-ventricular longitudinal function and cardiomyopathies [158], with the potential to become an increasingly incorporated imaging modality in routine clinical practice.

CMR allows for the accurate assessment of LV ejection fraction as well as chamber dilation and is a class 1 recommendation for the diagnosis of HF in patients with suboptimal TTE imaging (Table 2) [147]. CMR can provide greater details regarding anatomy and the type of cardiomyopathy, providing the detail required for diagnosis [50,146,153,159]. Compared to echocardiography, CMR has higher sensitivity and specificity for the diagnosis of hypertrophic cardiomyopathy and is also able to detect myocardial fibrosis (which creates a pro-arrhythmic substrate) via delayed myocardial enhancement [50].

##### Aortic Arch Atheroma

Aortic arch atheroma with a thickness of ≥4 mm is well known to be a significant risk factor for stroke recurrence (Figure 1) [36,40,73] and is found in approximately 1/3 of patients who have suffered an ischaemic stroke [32,33,34]. Alongside carotid artery disease and AF, severe aortic plaque is a major risk factor for embolic stroke, with severe plaque in the aortic arch seen via TOE having a one-year risk of stroke of 10% to 12% [160,161,162,163]. Amarenco et al.’s cohort study found that 28% of patients with undetermined stroke had aortic plaques measuring ≥4 mm, which was compared to 8% of patients with a known cause of stroke (*p* < 0.001), and that aortic atherosclerosis was an independent risk factor for ischemic stroke [73,164]. Patients with severe aortic arch atheroma (plaque > 5 mm) are also associated with higher rates of stroke and peripheral embolism [165,166]. Whilst aortic arch calcification is associated with plaque development and subsequent cardiovascular events [167,168], the French Study of Aortic Plaques in Stroke study found that plaques without calcification are also associated with an increased risk of recurrent stroke [169].

Whilst TTE can visualise the proximal ascending aortic aorta and aortic root, it struggles to accurately identify aortic arch atheroma [45]. Aortic arch atheroma is best detected using TOE, which has high sensitivity and specificity in detecting aortic arch atheroma (sensitivity of 75%; specificity of 84%), including with respect to various measurements such as ulceration, calcification, thrombus, and plaque thickness (Table 2) [45,164].

Cardiac CT and CMR can also assess aortic plaque structure (e.g., calcifications and fibrocellular tissue) and markers of instability (the size of the necrotic core and the presence of intraplaque haemorrhage) (Table 2) [51,52]. Additionally, 3D-multi-contrast CMR provides further details of plaque characteristics and morphology, including size, intraplaque haemorrhage, and superimposed thrombi [22,59]. Compared to TOE, it is less accurate in estimating plaque size but can better identify pseudoaneurysm formation, intraplaque haemorrhage, and penetrating ulcers [170].

In addition, plaque inflammation is associated with an increased risk of plaque rupture [40]. A PET scan can be used to identify plaque inflammation, hypoxia, and hypermetabolism, which are potential markers of plaque rupture [40]. There are currently limited data on the use of PET in assessing the risk of aortic plaque rupture and cardioembolic stroke, and whether its use for assessing aortic arch atheroma has clinical implications is still an evolving field.

CTA can also be used for aortic evaluation to identify aortic plaques, including location, size, and density, but not plaque mobility (Table 2) [45]. CTA can additionally visualise the distal ascending aorta, a location not visualised via TTE, and it can also detect vascular calcification [45]. TOE is superior to CTA with respect to aortic evaluation, with an accuracy of 84%, a sensitivity of 87%, and a specificity of 82% compared to TOE [171]. However, its specificity in detecting high-grade aortic arch atheroma is 99%, meaning that if the CTA demonstrates a negative result for high-grade atheroma, then the clinician can consider holding off with regard to TOE [172]. Overall, TOE is the gold standard for the detection of aortic arch atheroma [45], but CTA may be a good alternative to TOE for the evaluation of aortic arch atheroma depending on availability and the patient’s clinical status.

Notably, whilst the identification of aortic arch atheroma reveals whether a patient is at risk of stroke recurrence, there is currently no clear guidance on how this knowledge changes treatment. The Aortic-Arch-Related Cerebral Hazard (ARCH) prospective randomised trial compared aspirin plus clopidogrel vs. warfarin in patients with ischaemic stroke and aortic arch atheroma > 4 mm [173]. The trial found that aspirin plus clopidogrel resulted in a nonsignificant 24% reduction in stroke recurrence (*p* = 0.5) but significantly reduce rates of vascular death (*p* = 0.013) [173]. However, the trial was inconclusive due to its lack of statistical power, possibly contributed to by chance and the long duration of the trial (8 years) [173]. Overall, whilst aortic arch atheroma is a significant risk factor for stroke recurrence, its identification currently does not significantly change active management in current practice.

##### Cardiac Tumours

Primary cardiac tumours are rare, with a prevalence of 0.002–0.3%, and with >75% of these tumours being benign [174]. Cardiac tumour fragment detachment and a superimposed thrombus increase the risk of an embolic phenomenon, though the overall incidence of embolic stroke from cardiac tumours is low due to the overall low prevalence of primary cardiac tumours. Cardiac myxoma is the most common benign primary cardiac tumour, accounting for over 50% of primary cardiac tumours [174]. LA cardiac myxomas can give rise to embolic events in 30–40% of patients (Figure 1) [174]. TTE can be used to identify the majority of cardiac myxomas, though TOE is better for imaging right-heart myxomas (Table 2) [84].

Papillary fibroelastoma is the second-most-common primary cardiac tumour, with 80% being found on the cardiac valves [175]. They are usually small, being <20 mm in diameter, and they can often be mistaken for vegetations. However, fibroelastoma have a papillary structure and a homogenous speckled texture and are oval-shaped, with 50% having a stalk; in comparison, bacterial vegetations may have a changing appearance over time and are associated with other clinical signs of endocarditis (perivalvular abscesses, valvular destruction, valvular regurgitation, and clinically unwell patients) [176,177]. The sensitivities for detecting papillary fibroelastoma using TTE and TOE are approximately 62% and 77%, respectively [175]. Echocardiography can be used to identify these tumours via the identification of typical features, including their shape, well-demarcated borders, homogeneity, and stalk, with TOE being able to better identify smaller tumours [175]. Surgical excision of these tumours can be considered when there are no other identified causes of stroke [84].

However, echocardiography cannot be used to assess extracardiac extension and may not be able to characterise the tissue in enough detail. CMR can characterise tumour tissue with increased detail, including with regard to tissue composition and the extent of invasion [53]. A PET scan additionally allows for the metabolic characterisation of a tumour, aiding in tumour staging, the evaluation of distal metastases, and the evaluation of recurrence and response to therapy [53], and a combination of PET with CMR can be considered [53].

#### 3.2.2. Defects of the Atrial Septum

##### Patent Foramen Ovale (PFO)

PFO is found in 25–30% of the general population and in up to 40% in patients who have suffered a cryptogenic stroke [178,179,180,181]. PFO is a common cause of cardioembolic stroke, especially in younger patients (Figure 1) [35]. TTE, TOE, and transcranial doppler (TCD), combined with agitated saline contrast (“bubble study”), can be used for the identification of PFOs, with ≥3 microbubbles being seen in the left heart within three cardiac cycles being considered positive. Colour Doppler is sometimes used to enhance flow through the PFO [182].

Often, the initial study involves TTE due to its widespread availability and being better tolerated. Patients are also better able to comply with instructions for coughing and the Valsalva manoeuvre during TTE compared to TOE [183]. However, TOE has a higher detection rate for PFO compared to TTE and is also able to provide a more accurate shunt morphology (e.g., size, transseptal blood flow, and interatrial septum mobility), and it is the only diagnostic technique that can differentiate an intrapulmonary shunt from a PFO shunt (Table 2) [35,41,184]. Smaller shunts are more prone to false-negative results [185,186,187,188]. TOE remains the superior modality in imaging PFO, with its identification via TOE and subsequent closure resulting in lower rates of recurrent stroke compared to medication alone [189,190,191]. As such, patients < 60 years who are candidates for PFO closure are advised to undergo TOE, even if their TTE results are negative [192]. The limitations of TOE in PFO detection include the need for sedation, availability, and reduced Valsalva efficacy in microbubble shunting due to sedation [192].

TCD is also used for PFO detection (Table 2). It has fewer false negatives compared to TTE or TOE [192]. The corresponding sensitivities range from 91 to 100% [185,193,194,195,196,197,198,199], with specificities of 78–100% [187,193,194,197,198,199,200]. TCD may be more accurate than TTE or TOE in detecting smaller shunts [185]. Martínez-Sanchez et al. found that TCD identified twice the number of PFOs compared to TTE [201], and Tobe et al. found that TCD identified an additional 15% of PFOs that were missed when conducting TOE [186]. Meta-analyses comparing TCD and TOE found that TCD has the highest diagnostic accuracy compared to TOE and TTE (TCD: 94% sensitivity and 92% specificity; TOE: 89% sensitivity and 91% specificity; TTE: 45% sensitivity and 99% specificity) [184,202,203,204]. However, TCD is limited in that as an indirect technique, it is not able to provide anatomical information on PFO morphology and cannot identify whether a shunt is intracardiac or extracardiac [185]. TCD it is also not able to assess other potential cardioembolic sources [185]. Hence, TCD often needs to be combined with direct imaging of the PFO via TOE.

Overall, TTE, TOE, and TCD are all reasonable options for the detection of PFO. TCD has the highest sensitivity, with a wide range of sensitivities and specificities for TOE and TTE [192]. However, TOE remains the gold standard for PFO detection as TCD cannot identify the size or location of a shunt [205]. A combination of TOE and TCD is recommended to improve accuracy, especially for younger patients, with TOE being able to provide the anatomical detail that TCD cannot [182,203].

##### Atrial Septal Aneurysm (ASA)

An atrial septal aneurysm (ASA), diagnosed when the atrial septum is displaced by at least 10 mm from the midline, is a potential risk factor for ischaemic stroke (Figure 1) [206]. These aneurysms are formed by interatrial pressure differences or primary septal malformations and are often found associated with other defects such as mitral valve prolapse (MVP), PFO, and atrial septal defects (ASD) [207]. Pearson et al. found that ASA occurred in greater frequency in patients with cryptogenic stroke [208], with a meta-analysis of case–control studies finding that in stroke vs. non-stroke patients < 55 years old, the odds ratio of stroke was 6.1 (95% CI, 2.5 to 15.2) for ASA, with larger ASA size also having a stronger association with cryptogenic stroke [209]. The PFO-ASA study also found that ASA is strongly associated with PFO [180]. Whilst the association between PFO, ASA, and ischaemic stroke is well-established for patients < 55 years old, their association with patients > 55 years old is not as clear. This is likely contributed by other risk factors for stroke that are more common among older patients [209]. The association of ASA with cardioembolic stroke is likely secondary to associated interatrial shunt/PFO and subsequent paradoxic embolisation, as well as primary thrombus formation within the aneurysm. Interestingly, Mügge et al. found that variation in ASA morphology (length, bulging, and oscillations) did not affect the rate of embolic events. This suggests that the association between ASA and cardioembolic stroke may be secondary to its associated cardiac defects rather than being a direct source of embolism [207].

TTE may be the initial screening tool for most patients; however, TOE is superior to TTE in imaging the inter-atrial septum and therefore detecting ASA (Table 2) [206,207,210]. TOE can be used to better characterise atrial septum morphology compared with TTE, with one study finding that TTE missed the presence of ASA in 47% of patients [207]. In comparison, TOE has a sensitivity of 90–100% and a specificity of 98–100% [23]. With an addition of colour flow doppler or an agitated saline contrast study, associated right-to-left shunts can be detected. Overall, in cases of suspected PFO/ASA, TOE should be considered a gold standard for diagnosis.

#### 3.2.3. Valvulopathies

##### Infective Endocarditis (IE)

Approximately 10% of patients with IE suffer an embolic stroke, with the risk of stroke being highest prior to and in the first two weeks of antibiotic therapy [19,211]. Embolic phenomena are among the most common complications of IE, especially if the mitral and aortic valves are involved (Figure 1). Mobility, consistency, distribution, and dimensions of vegetations affect embolic risk, with increased vegetation size being associated with an increased risk of embolism [212].

The American Heart Association’s guidelines for the management of IE recommend using TTE as the initial imaging modality for suspected IE (Table 2) [211]. TTE’s overall sensitivity in detecting IE is only 62–79%, with a sensitivity of 20–40% for left-heart IE [19] and 85% for tricuspid valve IE [211]. Among patients for whom there is a high clinical suspicion of IE and negative or inconclusive TTE results, TOE can be used to increase the detection rate of IE to 85–90%, and it is especially sensitive in prosthetic valve IE and in detecting complications such as abscesses and leaflet tears [48]. Especially for patients with a suspected abscess, TOE should be used, offering a sensitivity of 90% vs. TTE’s 50% [48,213]. TOE is also useful in cases of perforated MV secondary to an infected aortic valve’s regurgitant jet [214]. However, small anterior abscesses are better seen via TTE. As such, TTE alone may suffice for patients with high-quality negative TTE results and low clinical suspicion of IE; however, both TTE and TOE should be used for patients with suspected perivalvular involvement [48]. Notably, echocardiography is not 100% specific or sensitive for IE, and up to 15% of patients with IE may have a negative echocardiogram (very small vegetations, pre-existent lesions (e.g. MVP), degenerative lesions, prosthetic valves, atypical locations) [48]. Echocardiography should be repeated if clinical suspicion remains high.

Cardiac CT and CMR can also be used, particularly for assessing complications such as paravalvular abscesses or pseudoaneurysms (Table 2) [48,49]. Cardiac CT has a 97% sensitivity and 88% specificity in detecting IE [49]. Whilst CMR can be used to detect IE-related cardiac complications such as perivalvular abscesses and regurgitation, the temporal resolution of CMR is lower than that of TOE, limiting CMR’s role in visualising vegetations [215]. As such, there are currently no large studies on CMR’s diagnostic role in IE, though it may be a useful addition to but not a replacement for echocardiography [49,60,61].

PET/CT can be used to diagnose IE via the higher metabolic activity in inflammatory and infected tissue, especially in inconclusive cases (Table 2) [68]. PET/CT has been shown to lead to a change of therapy for 35% of patients and is especially sensitive in cases of device-related infections [49,216]. However, PET/CT carries a risk of false positives and negatives, contributed by antibiotic use, small vegetation size, recent cardiac procedures, and patient factors (a lack of compliance with a low-carbohydrate diet; elevated serum glucose levels) [216]. Another modality to consider in cases of diagnostic uncertainty is leucocyte scintigraphy with SPECT/CT, as it has high specificity for infection due to granulocyte recruitment to the site of infection [217,218,219,220]. Leucocyte scintigraphy with SPECT/CT can also be used for prognostication, with a positive test being associated with high infectious activity and a poor prognosis [218]. Its main limitations are as follows: four patient visits are required, it has a lengthy preparation time, and it poses a risk of missing small infectious foci [49].

Overall, TTE should be first used in cases of suspected IE, with additional TOE in cases of suspected perivalvular involvement or inconclusive cases. To date, there are no direct comparisons between TOE vs. PET-CT for diagnosing IE; however, PET/CT and leucocyte scintigraphy with SPECT/CT can be considered in cases of inconclusive echocardiograms [37].

##### Prosthetic Valve Endocarditis and Thrombi

Prosthetic valves increase the risk of ischaemic stroke due to their association with IE and thrombus formation; prosthetic valve thrombus is detected in 12–40% of registries (Figure 1) [206,221]. A study by Puvimanasinghe et al. reported a significantly higher incidence of ischemic stroke among patients with mechanical valves compared to those with bioprosthetic valves [222]. The rates of prosthetic valve thrombosis are especially high for the mitral and right-sided valves [222,223,224]. As such, the risk of stroke for patients without anticoagulation can be as high as 4% per year among patients with mechanical valves and 1.3% per year among those with bioprosthetic valves [225,226].

Optimally, both TTE and TOE should be used for the comprehensive imaging of prosthetic valves. Bioprosthetic prosthetic valve thrombosis is diagnosed when there is a 50% rise in prosthesis gradient within 5 years post-implantation, increased cusp thickness, or atypical cusp movement that responds to anticoagulation (e.g., a 50% reduction in prosthesis gradient) [227,228,229]. Overall, TOE provides better visualisation of prosthetic valves compared to TTE; Werner et al. found that TOE was superior to TTE in identifying prosthetic valve endocarditis (*p* < 0.001) and prosthetic valve thrombi (*p* < 0.01) (Table 2) [230]. Overall, TOE had a sensitivity of 86% and a specificity of 88% in detecting prosthetic valve abnormalities, which can be compared to the same values for TTE of 57% and 63%, respectively [230]. For both aortic and mitral prosthesis, TOE was superior to TTE in detecting abnormalities [230]. This difference is likely contributed by TTE’s restricted acoustic views combined with the echogenic properties of prosthetic valve materials, making subtler anomalies like small vegetations and thrombi harder to detect using TTE. In addition, TOE provides an unobstructed view due to the proximity of the oesophagus to the heart as well as higher-frequency transducers allowing for the visualisation of smaller masses and the better visualisation of the device’s atrial surface [230]. Colour doppler can improve the anatomical information obtained; allow for the detection of prosthetic complications such as paravalvular leaks and pinhole defects; and be used to measure pressure gradients for valve degeneration [231,232,233]. Additionally, 3D TOE can also be used to augment this information, with good accuracy in detecting defect size and location as well as regurgitation severity and location [47].

If the results of echocardiographic imaging are suboptimal, cardiac CT can be used, especially in complex cases (e.g., multiple prostheses, valve-in-valve procedures, etc.) for more detailed tissue characterisation of leaflet calcification, thickening, and thrombus (Table 2) [47]. Cardiac CT can also assess right-sided structures, though there are limited data on its use in identifying right-sided prosthetic valve dysfunction [234,235]. CMR with phase contrast velocity mapping can be used to quantify and precisely locate regurgitant jets better than when using an echocardiogram and can be especially useful in the case of suboptimal quantification of regurgitant jets via TOE [57].

PET/CT can be used for the diagnosis of prosthetic valve IE, with an increased metabolic uptake in valvular infection and inflammation (Table 2) [236]. A large meta-analysis found that PET/CT has a sensitivity of 80% and a specificity of 73% in diagnosing prosthetic valve IE [237]. In addition, the recent prospective cross-sectional study by Bing et al. on patients with bioprosthetic valves found that radioisotope uptake was higher in thrombi and that its uptake regressed with anticoagulation. As such, PET/CT has the potential to both identify valvular thrombosis as well as monitor the efficacy and progression of thrombus formation with therapy [238].

##### Mitral Valvulopathy

Mitral stenosis (MS), usually caused by a previous affliction with rheumatic fever, increases the risk of ischaemic stroke, with the Framingham study finding that MS (irrespective of AF) is associated with a risk of stroke corresponding to 4.2/100 patient years (Figure 1) [239]. Endothelial damage, blood stasis secondary to LA dilatation and loss of atrial systole, and a hypercoagulable state produced by the increased release of prothrombotic mediators result in increased thrombogenicity, which increases the risk of AF and ischaemic stroke [240]. Mitral regurgitation (MR) is associated with increased rates of HF [241], with HF being one of the components of the CHA2DS2-VASc score for stroke risk stratification for AF [242]. Handke et al. found that the prevalence of LA thrombi was 27% in patients with MR, which was associated with a significantly higher risk of embolic events [243]. However, significant MR may in fact protect against LA spontaneous echo contrast in patients with non-rheumatic AF [244], with other studies finding that MR is neither an additional risk factor nor a protective factor for thromboembolic events in patients with AF [245,246,247]. Mitral valve prolapse (MVP) may be associated with an increased risk of ischaemic stroke, with studies including the Framingham Heart Study suggesting that individuals with MVP may have a slightly elevated risk of embolic events, including stroke [239,248,249,250]. However, this is largely driven by the increased incidence of AF in this population [250,251]. Other studies involving younger patients have not found that MVP is associated with ischaemic stroke, with another study finding that MVP-associated MR is protective against stroke [249,251,252]. Overall, MS is associated with an increased risk of ischaemic stroke, whilst the relationship between MR, MVP, and stroke is less clear. As such, anticoagulation is indicated for MS associated with AF, LA thrombus, and previous embolic events.

The assessment of MS severity is crucial, with severe MS associated with increased prevalence of AF and therefore increased thromboembolic risk [253]. TTE and TOE provide good assessments of the physiology and anatomy of the MV (Table 2). Continuous wave doppler from apical TTE or mid-oesophageal TOE windows are used to estimate MV gradients, closely corresponding to the MV gradient measured upon catheterisation [254]. Trans-mitral gradients, alongside MV area planimetry, pressure half-time, and flow velocities, are used to assess stenosis severity [255]. Other echocardiographic features of severity include commissural fusion, leaflet thickening, calcification, and mobility [255]. TOE should be considered if there is a suspicion of intracardiac thrombus associated with MS [83]. CMR can be used to better quantify associated LA dilatation, which is associated with LA thrombus [256]. With greater reproducibility, CMR can hence be considered for patients with suboptimal echocardiography views, though it may not be able to visualise torn cordae (better seen via echocardiography) or calcification (better seen via CT) [257].

##### Mitral Annulus Calcification and Global Cardiac Calcification

Mitral annulus calcification (MAC) is caused by lipid and calcium deposition in the mitral valve annular fibrosa, with a multifactorial aetiology including an atheroslecrosis-like inflammatory process, hypertension, hyperlipidemia, obesity, smoking, diabetes mellitus, and aortic stenosis [258]. MAC is associated with a greater risk of ischaemic stroke, with some studies finding that mitral annulus calcification is an independent predictor of ischaemic stroke (Figure 1) [259,260]. Some studies, including the Framingham Heart study, have found that MAC is associated with an increased risk of ischaemic stroke, possibly due to its association with AF from LA enlargement and conduction system defects [261,262,263,264]. Others found that this association was not significant after adjusting for confounders [265,266]. The association between MAC and ischaemic stroke risk could instead reflect general atherosclerotic risk rather than direct causation or associated factors (e.g., inflammatory, metabolic, and haemostatic risk factors) increasing the risk of stroke [261,264,267]. In addition, Li et al. found that higher global cardiac calcium scores, which can be used to quantify cardiac calcium burden, are associated with increased rates of AF and recurrent ischaemic stroke [268]. Calcium deposits themselves may act as a source of thromboembolism due to turbulent flow across a diseased valve resulting in shear stress on the annular calcium [268]. Cardiac calcium burden may also reflect a shared pathological process with atrial cardiopathy as well as cerebrovascular atherosclerosis, including shared atherosclerotic risk factors such as diabetes, hyperlipidaemia, hypertension, and age [269,270]. This suggests that a heavy cardiac calcium burden is associated with a high-risk phenotype for ischaemic stroke [268].

MAC can be evaluated via TTE, which can be used to assess the thickness/severity of cardiac calcification at the mitral annulus, submitral apparatus, and papillary muscle (Table 2) [258]. Doppler echocardiography can also be used to measure changes in flow velocity (which may be increased in severe stenosis) to indirectly assess the progression of MAC, though it does not directly assess MAC itself [258]. TOE can be used to provide an additional assessment of the mechanism and severity of associated valve dysfunction, with 3D echocardiography being able to better map out the valve [271]. TOE can be used if TTE windows are suboptimal and can also differentiate between calcification, infection, thrombi, and infection [272]. Notably, there is no standardised echocardiography grading system for MAC severity.

For a more accurate and comprehensive assessment of calcium scores associated with MAC, cardiac CT can be considered (Table 2). Cardiac CT provides better quantitative and qualitative data on calcification severity through calculation of the calcium score via the Agaston method, and also assess for special features such as caseous MAC or LV outlet tract extension [272]. Overall, cardiac CT provides the best overall assessment of global cardiac calcification and has improved spatial resolution for MAC compared to TTE, though it cannot be used for the quantification of MR/MS or to calculate trans-mitral gradients [271,272].

##### Aortic Valvulopathy

Aortic stenosis (AS) and aortic regurgitation (AR) are associated with an increased risk of ischemic stroke (Figure 1). Aortic valve disease can lead to alterations in blood flow patterns, causing turbulence and changes in shear stress within the aorta [273,274]. This can promote atherosclerotic plaque formation in the ascending aorta, which may embolize and result in ischemic stroke [275]. In severe AS, the rates of ischaemic stroke range from 5.6–21.8 per 1000 patient years and are associated with increased mortality [276,277,278]. The Tromsø Study found that AS was an independent risk factor for ischemic stroke, with an associated increased risk of stroke even in mild–moderate stenosis [279]. The SEAS (Simvastatin and Ezetimibe in Aortic Stenosis) trial found an increased event rate with increasing severity of AS [280]. Moreover, significant AS is associated with poorer functional outcomes post-stroke, which may be contributed by fixed LV outflow tract obstruction and reduced cardiac output resulting in cerebral hypoperfusion, shared cardiovascular risk factors, and increased peripheral vascular resistance [281].

In addition, moderate–severe AR is associated with an increased risk of stroke due to LV structural changes, changes in haemodynamics, and increased thromboembolic potential [282,283]. Severe AR can result in retrograde flow in the descending aorta [283]. This results in altered shear stress and flow patterns, potentiating aortic atherosclerosis and complex aortic plaque formation and therefore increasing the risk of ischaemic stroke [283].

TTE has high sensitivity (80–90%) and specificity (90–95%) in diagnosing AS and AR (Table 2) [163]. Compared to TTE, TOE has higher sensitivity (>90%) and specificity (approaching 100%) in the assessment of AS and AR [284].Cardiac CT and CMR can also be utilised to visualize aortic stenosis, providing detailed anatomical information and aiding in the assessment of disease severity [163]. Pawade et al. reported that aortic valve calcium scoring derived from cardiac CT strongly correlates with the severity of AS and is predictive of clinical outcomes, offering robust specificity (90%) but moderate sensitivity (70%) for disease detection [285]. While CMR can reliably assess AS and AR severity, it tends to be slightly less sensitive than echocardiography, with studies showing a sensitivity ranging from 70% to 85% and specificity levels exceeding 90% [163].

**Table 2:** Summary of common imaging modalities in relation to their use in identifying an individual cardioembolic source. ‘*’ = first line, ‘◊’ = gold standard, ‘+’ = poor diagnostic performance, ‘++’ = reasonable diagnostic performance, and ‘?’ unclear diagnostic performance.

	TTE	TOE	CT	CMR	Nuclear	Others
LA dilation and LA thrombus	* + First-line investigation for cases of AF [80] Limited views of the LAA [80] Low sensitivity for LA thrombus [80]	◊ Technique of choice for detecting posterior cardiac structures including atria and appendages [83] Sensitivity of 93%, specificity of 100%, and accuracy of 99% [83] Markers include spontaneous echo contrast and measures of LAA mechanical dysfunction [80]	++ Assesses LAA size and morphology [87] Higher false-positive rate in patients with LAA stasis [87]	++ Assesses LAA size and morphology [89] Higher false-positive rate due to lower spatial resolution and its susceptibility to slow flow [89]		Strain imaging Independently associated with ischaemic stroke [95,96] Able to identify patients with acute embolism [94] Predicts post-stroke mortality [94] Can predict subclinical AF [97] Holds potential for improving risk stratification among patients with cryptogenic stroke
LV thrombus	* ++ Specificity of 85–90%, sensitivity of 95% [9,115] 10–46% of TTEs are inconclusive due to difficulty in visualising the LV apex [116,117]	++ Superior in terms of imaging unclear LV echogenic structures and apex [80,119,120] Inferior to TTE in terms of evaluation of LV thrombus [80,119,120]	++ Approximately the same specificity and sensitivity compared to TTE [121]	◊ More accurate than TTE and TOE [25,54,55,56] Thrombi can be further characterised with LGE-CMR [9] LGE CMR—higher sensitivity and specificity compared to TTE [53] Can identify structural features that increase risk of LV thrombus (e.g., myocardial scar burden, infarct size) [53] Limited evidence directly comparing CMR to TOE in detection of LAA thrombus [53]		
HF/Cardiomyopathy	* ++ Allows for assessment of reduced ejection fraction that increases risk of cerebral hypoperfusion [147,148] Visualises wall motion abnormalities and dilated LA or LV (incomplete ventricular emptying and blood stasis) [147,148]	++ Allows for the evaluation of intracardiac thrombi and spontaneous echo contrast Better than TTE in detecting LA thrombus [148,152] Not included in current guidelines for HF diagnosis Strongly recommended for patients with cardiomyopathy and AF or who have suffered a cryptogenic stroke in order to screen for intracardiac thrombi [146]	++	◊ Class 1 recommendation for diagnosis of HF in patients with suboptimal TTE [147] Allows for accurate assessment of ejection fraction and chamber dilation [147] Able to detect myocardial fibrosis (which creates a pro-arrhythmic substrate) via delayed myocardial enhancement [50]		Speckle tracking/GLS LV deformation [153] Reliable marker for arrhythmias in non-ischaemic cardiomyopathy [157] Limited in patients with suboptimal acoustic windows and AF Incorporated into the 2017 European Association of Cardiovascular Imaging guidelines for the evaluation of left- and right-ventricular longitudinal function and cardiomyopathies [158]
Aortic arch atheroma	+ Unable to identify aortic arch atheroma [45]	◊ Sensitivity: 75%, specificity: 86% [45,164]	++ Allows for assessment of aortic plaque structure and markers of instability [51,52] Less accurate in estimating plaque size compared to TOE [170] Better at identifying pseudoaneurysm formation, intraplaque haemorrhage, and penetrating ulcers [170]	? Tracks inflammation, hypoxia, neoangiogenesis, and calcification, which are potential markers of plaque rupture [40]	++ CT angiography Good alternative to TOE [45] Can identify aortic plaques [45] Can visualise distal ascending aorta [45] Unable to evaluate plaque mobility [45]	
Cardiac tumours	+ Able to identify the majority of cardiac myxomas [84] Sensitivity for papillary fibroelastoma: 62% [175]	++ Better than TTE for imaging right-heart myxomas [84] Sensitivity for papillary fibroelastoma: 77% [175]	++	◊ Characterises tissue composition and extent of invasion [53] Can be combined with PET to streamline investigations [53]	Metabolic characterisation—aids in tumour staging, evaluation of distal metastases, evaluation of recurrence, response to therapy [53] Can be combined with CMR to streamline investigations [53]	
PFO	* + Better compliance for coughing and valsava [183]	◊ Ideally combined with TCD to improve accuracy [182,203] Higher detection rate for PFO compared to TTE [35] More accurate shunt morphology [41] Can differentiate an intrapulmonary shunt from a PFO shunt [184] Smaller shunts are more prone to false-negative results [185,186,187,188] Reduced Valsalva efficacy in microbubble shunting due to sedation [192]	+	+		TCD ◊ Ideally combined with TOE for shunt location and morphology [182,203] Highest diagnostic accuracy [184,202,203,204] Fewer false negatives than echocardiography [192] Sensitivity 91–100%, specificity 78–100% [187,193,194,197,198,199,200] More accurate in detecting smaller shunts Indirect technique: cannot identify location of shunt, whether intra- or extra-cardiac [205]
ASA	* Missed diagnosis for 47% of patients [207]	◊ Offers more detailed characterisation of atrial septum [206,207,210] Sensitivity of 90–100%, specificity of 98–100% [23]				
IE	* ++ Sensitivity of 62–79% [19] Native valve IE: sensitivity—83%, specificity—84% [230] Left heart IE: sensitivity 20–40% [19] Right heart IE: sensitivity—85% [211] Better at visualising small anterior abscesses [48] May suffice for patients with high-quality negative TTE results and low clinical suspicion of IE [48]	◊ Useful for patients with high clinical suspicion and negative/inconclusive TTE [48,213] Detection rate: 85–90% [48] Especially good for prosthetic valve IE [48] Good for the detection of complications (abscesses, leaflet tears); sensitivity for abscess: 90% vs. TTE’s 50% [48,213] Both TTE and TOE should be used for patients with suspected perivalvular involvement [48] Up to 15% may have a negative echocardiogram; echocardiogram should be reobtained if clinical suspicious remains high [48]	++ Can be used to assess complications [48,49] Sensitivity: 97%, specificity: 88% [49]	? Can be used to assess complications; however, there are no large studies investigating its diagnostic role in IE [215]	No direct comparisons between TOE vs. PET-CT Can be considered in cases of inconclusive echocardiograms [37] PET Leads to change of therapy in 35% of patients [49,216] Sensitive in device-related infections [49,216] Risk of false positives and negatives [216] Dependence on patient factors (lack of compliance to low-carbohydrate diet, elevated serum glucose level) [216] Leucocyte scintigraphy with SPECT/CT High specificity for infection, able to prognosticate [217,218,219,220] Requires four patient visits, lengthy preparation, may miss small infectious foci [49]	
Prosthetic valve IE and thrombi	* ++ Both TTE and TOE should be used [227,228,229] Offers good visualisation of the LV [230] Limited view of LA [230] Sensitivity: 57%, specificity: 63% for IE [230]	◊ Both TTE and TOE should be used [227,228,229] Better evaluation of LA and left-sided valves [230] Limited views of LV [230] Superior to TTE for prosthetic valve IE and thrombi [230] Sensitivity: 86%, specificity: 88% for IE [230] Colour doppler can evaluate paravalvular leaks and pinhole defects and measure pressure gradients [231,232,233]	++ Useful for complex cases [47] Better tissue characterisation Allows for the assessment of right-sided structures [47] CMR with phase contrast velocity mapping can be used to quantify and precisely locate regurgitant jets more effectively than echocardiography [57]	Increased metabolic uptake in valvular infection and inflammation [236] Sensitivity of 80% and specificity of 73% for IE [237] Potential to monitor efficacy and progression of thrombus formation with therapy [238]		
MS	* ++ Allows for the assessment of MS severity, which is associated with AF MV gradients, area planimetry, flow velocities used to assess stenosis severity [255] Commissural fusion, leaflet thickening, calcification, and mobility associated with stenosis severity [255]	◊ MV gradients assessed via continuous wave doppler from mid-oesophageal TOE [254] Better in inconclusive cases [254] Should be considered if there is a suspicion of intracardiac thrombus associated with MS [83]	++	++/? Better at quantifying ventricular volume and myocardial mass [257] Greater reproducibility [257] May not be able to visualise calcification/torn chordae [257] Limited data on use of CMR in MV evaluation		
MAC/Cardiac calcification	* (for MAC) ++ TTE with doppler for measuring global calcium scores, which are associated with ischaemic stroke [258] Assesses severity of cardiac calcification [258]	++ Provides additional assessment of mechanism and severity of associated valve dysfunction [272] Useful in suboptimal TTE windows [272] Can differentiate between calcification, infection, thrombus and infection [272]	* (for global calcification) ◊ Better quantitative and qualitative data on calcification severity [272] Calculates the calcium score via the Agaston method [272] Scans for special features such as caseous MAC or LV outlet tract extension [272]			
AS/AR	* Sensitivity: 80–90%, specificity: 90–95% [163] Identification of severe AR that alters shear stress and increases risk of aortic atherosclerosis and subsequently stroke [163] Measurement of AS severity, which is associated with increased stroke rate Visualises fixed LV outflow tract obstruction that may reduce cardiac output and cause cerebral hypoperfusion [281]	◊ Sensitivity: >90%, specificity: almost 100% [284]	++ Sensitivity: 70%, specificity: 90% [285] Assesses disease severity [163]	++ Sensitivity: 70–85%, specificity: > 90% [163] Assesses disease severity [163] Generally less sensitive than echocardiography [163]		-

## 4. Conclusions

In conclusion, cardiac imaging plays a crucial role in identifying cardioembolic causes of stroke, and the choice of imaging modality should be tailored to the individual patient based on their specific circumstances and potential risk factors, taking into consideration resource allocation, availability, and logistical, financial, and clinical factors. Echocardiography is the mainstay of cardiac evaluation. TTE is the first line in the basic cardiac evaluation of most cardioembolic causes of stroke, including LA dilatation, LA thrombus, LV thrombus, evaluation for HF and potential cardiomyopathy, atrial septal defects (ASA and PFO), IE, prosthetic valve thrombus, mitral annulus calcification, and valvular disease (MS, AS, and AR). It can be used to measure chamber size and systolic/diastolic function and is readily available and non-invasive. TOE is the gold standard for evaluating LA dilatation and thrombus, aortic arch atheroma, PFO, ASA, MS, IE, prosthetic valve thrombus, and the aortic valve. TOE is also superior to TTE in detecting posterior cardiac structures including atria and appendages. It should be strongly considered, especially for patients whose TTE results are inconclusive, and clinical suspicion for the above potential cause is high. However, its risks vs. benefits must be weighed and considered on an individual basis, in view of its semi-invasive nature and minor procedural risk. Cardiac CT and CMR provide better soft tissue characterisation, high-grade anatomical information, spatial and temporal visualisation, and image reconstruction in multiple planes and are useful in inconclusive echocardiograms. Cardiac CT is the gold standard in evaluating global calcification, and CMR is the gold standard in evaluating LV thrombus, HF, cardiomyopathy, and cardiac tumours. Their use is mainly limited by resource allocation, availability, radiation exposure, contrast risk, and cost. Nuclear imaging is not routinely used but can be considered when looking for systemic causes of a pro-thrombotic phenotype, such as cancer. Emerging data also suggest that nuclear imaging can be used to increase diagnostic accuracy and localisation of IE, and in identify aortic plaques at high risk of rupture. Overall, cardiac imaging plays a critical role in diagnosing cardioembolic causes of stroke, and the choice of imaging approach should be tailored to the individual patient.

## Data Availability

No new data were created or analysed in this study. Data sharing is not applicable to this article.
